# Ultrasound Instead of X-Ray to Diagnose Neonatal Fractures: A Feasibility Study Based on a Case Series

**DOI:** 10.3389/fped.2022.847776

**Published:** 2022-05-26

**Authors:** Jing Liu, Li Zhang, Ru-Xin Qiu

**Affiliations:** ^1^Department of Neonatology and NICU, Beijing Chaoyang District Maternal and Child Healthcare Hospital, Beijing, China; ^2^Department of Neonatology and NICU, Beijing Chao-Yang Hospital West Branch, Capital Medical University, Beijing, China

**Keywords:** ultrasound, X-ray, neonates, fracture, image

## Abstract

**Background:**

Fracture is a common birth injury in neonates, and its diagnosis mainly depends on chest X-ray examination, while ultrasound is typically not included in the diagnostic work-up of neonatal fractures. The aim of this study was to investigate the feasibility of using ultrasound to replace X-rays for the diagnosis of fractures in newborns and to determine the ultrasound characteristics of such fractures.

**Methods:**

Bedside ultrasound with an appropriate probe and scanning angle was performed on 52 newborn infants with suspected fractures based on physical examination findings, and the ultrasound results were compared with the X-ray examination results.

**Results:**

All 52 infants (100%) showed typical signs of fracture on ultrasound, including 46 cases of clavicle fracture, 3 cases of skull fracture, 2 cases of rib fracture, and 1 case of humerus fracture. Ultrasound was able to detect interrupted cortical continuity, displacement or angulation at the broken end, and callus formation during the recovery period. Chest X-ray examination was performed on 30 patients and identified 96.7% (29/30) of fractures, and the coincidence rate between ultrasound and X-ray was 100%. However, the sensitivity of ultrasound was higher than that of X-ray.

**Conclusion:**

Ultrasound diagnosis of neonatal fracture is accurate, reliable, simple, and feasible. Therefore, it can replace X-ray examinations for the routine diagnosis of common types of neonatal bone fractures.

## Introduction

Fracture is a common birth injury in neonates (1), and the incidence of clavicle fracture ranges from 0.2 to 4.4% in foreign reports (2, 3) and from 0.14 to 0.46% in developing regions (4, 5). For a long time, the standard diagnostic treatment for bone fracture in neonates to determine the severity and treatment or recovery time has been X-ray due to its low cost, high speed, wide availability, and ease of use, while ultrasound is typically not included in the diagnostic work-up of neonatal bone fractures. However, X-ray examination has many disadvantages, such as the need to move the infant, which has the potential to aggravate the fracture, the amount of time required, the increased radiation exposure not only to the patient himself but also to other infants within the same wards and medical staff, the possibility of missed diagnosis, etc. Therefore, it is of great significance to find a simple and reliable new method that can replace the X-ray diagnosis of neonatal fractures. Recently, ultrasound has been used to diagnose Salter-Harris I fractures of the distal humerus, a rare kind of fracture in neonates (6). The purpose of this study was to explore the feasibility and reliability of using ultrasound to replace X-rays to diagnose fractures in newborns.

## Subjects and Methods

### Subjects

This study protocol was approved by the Ethics Committee of Beijing Chaoyang District Maternal and Child Healthcare Hospital (No. 2011-LC-Ped-01), and all methods were performed in accordance with the relevant guidelines and regulations. Written informed consent was obtained from the patients' parents.

From January 1, 2018 to October 20, 2020, about 52 cases of combined born fracture were identified among hospitalized patients. The diagnostic criteria were based on physical examination findings, including local swelling and pain, bilateral asymmetry of the clavicle, local tenderness, and bone crepitus (the early stage of fracture) or callus formation (the late stage of fracture). The exclusion criteria were no bone crepitus in the early stage and no callus formation in the late stage during physical examination. Sonograms of the contralateral bones of these patients were used as controls.

### Methods

#### Instruments and Examination Methods

**Figure 1:**
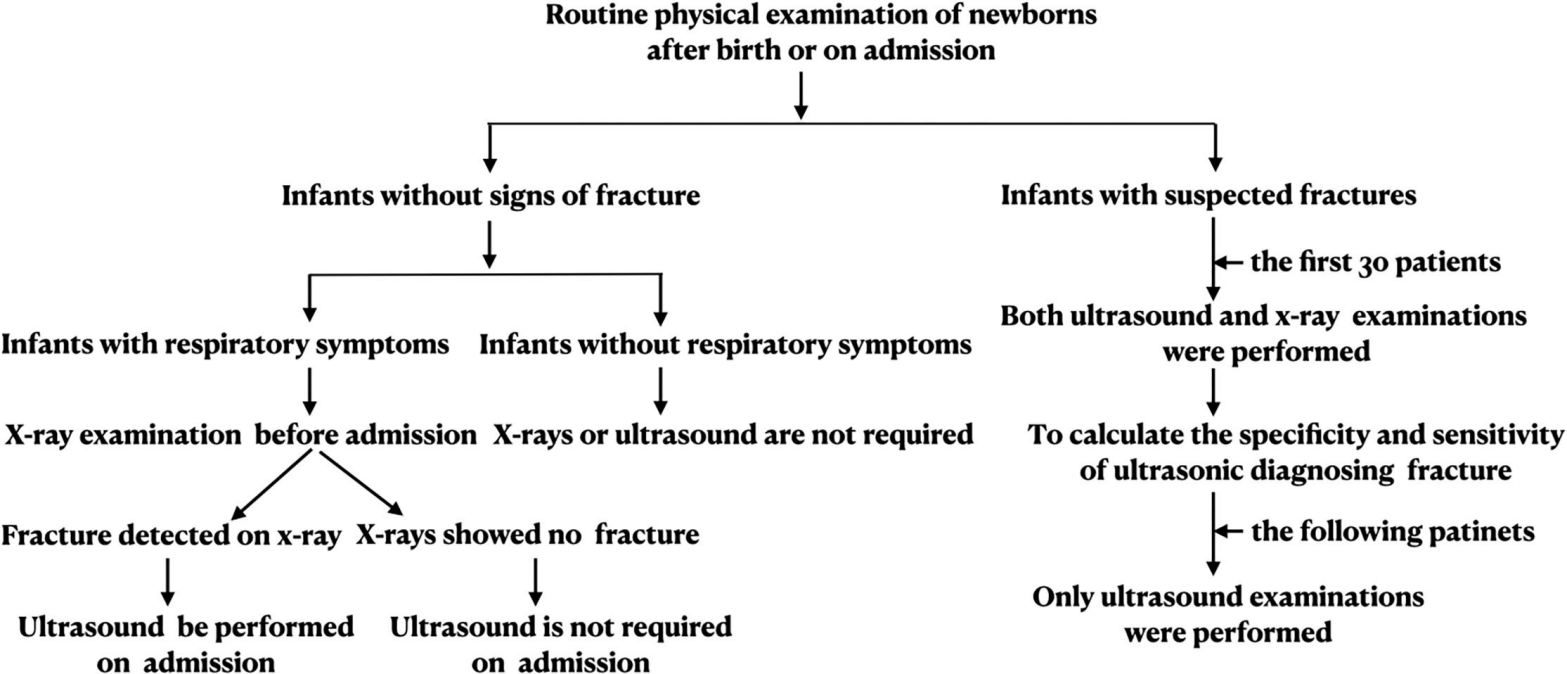
Flow chart of the use of ultrasound to diagnose neonatal clavicle fracture. Flow chart of ultrasonographic diagnosis of clavicle fracture, refer to this process for diagnosis of other types of fracture.

A Voluson S10 (GE Healthcare, USA) ultrasound system with a 9-L linear array probe or an EPIQ5 (Philip Healthcare, Netherlands) ultrasound system with a linear array probe (frequency of 4–12 MHz) was used for bone ultrasound examination in this study. The infant was kept in a quiet state during the examination. If a clavicle fracture was suspected, the infant was kept in the supine or lateral position (with the injured side facing up), and the infant's head was gently turned to the unaffected side to facilitate the ultrasound operation. If an upper or lower limb fracture was suspected, the infant was kept in the supine position so that the limb was in a natural position. At the beginning of the examination, the probe was kept at an angle or perpendicular to the bone examined and then rotated in a clockwise or counterclockwise direction. Fracture signs could be found as the probe gradually became parallel to the examined bone. If a rib fracture was suspected, then the infant was kept in the prone position, and the probe was kept parallel to the rib when scanning. All the ultrasounds were performed by one trained neonatologist. The flow chart of the use of ultrasound to diagnose neonatal fracture is shown in Figure 1 (taking clavicle fracture as an example).

#### Observation Indicators

The observation signs of fracture on ultrasound in this study, including intact continuity of the cortical bone, the presence of a broken end, fracture line, angulation, ectopy or displacement, and the presence of callus formation during the recovery period, were determined during the examination.

#### X-Ray Examination

In this study, data from 30 infants with clavicle fractures, who underwent both chest X-ray and ultrasound examination, were used to calculate the sensitivity and specificity of ultrasound for the diagnosis of clavicle fractures.

### Statistical Analysis

The sensitivity and specificity of ultrasound diagnosis of fracture were determined.

## Results

### General Information of the Two Groups

#### General Information

The gestational age of the patients ranged from 30^+~^ to 40^+5^ weeks, including 7 preterm infants and 45 full-term infants. There were 31 boys and 21 girls. There were 39 first births from the first pregnancy and 13 second and subsequent births. There were 39 cases of vaginal delivery (including 9 cases of forceps delivery) and 13 cases of cesarean section. The birth weight ranged from 1,370 to 4,510 g, of which 41 cases (78.8%) were >3,500 g.

#### Cause of Hospitalization and Time to Fracture Diagnosis

There were 16 cases of severe jaundice and hemolytic disease of the newborn,12 cases of intrauterine pneumonia (including 2 cases with persistent pulmonary hypertension and 2 cases with pulmonary hemorrhage), 8 cases of respiratory distress syndrome (including 2 cases with persistent pulmonary hypertension), 5 cases of meconium aspiration syndrome, 4 cases of coagulation disorders and disseminated intravascular coagulation (one case with pulmonary hemorrhage), 5 cases of severe asphyxia and hypoxic-ischemic encephalopathy, 2 cases of metabolic bone disease in preterm babies, and 2 cases of cranial hematoma. All infants were found to have suspected fracture on physical examination at the time of admission and were diagnosed by ultrasound, and 30 cases were confirmed by chest X-ray. The earliest admission time was 22 min after birth, and the latest was 10 days after birth.

#### Fracture Types

Of the 52 cases of fractures in this study, 50 were caused by birth injuries. There were 46 cases of clavicle fracture (19 on the left side and 27 on the right side), 3 cases of skull fracture, 2 cases of rib fracture (considered metabolic bone disease), and 1 case of humerus fracture (right side).

### Ultrasound Findings of Neonatal Fractures

**Figure 2:**
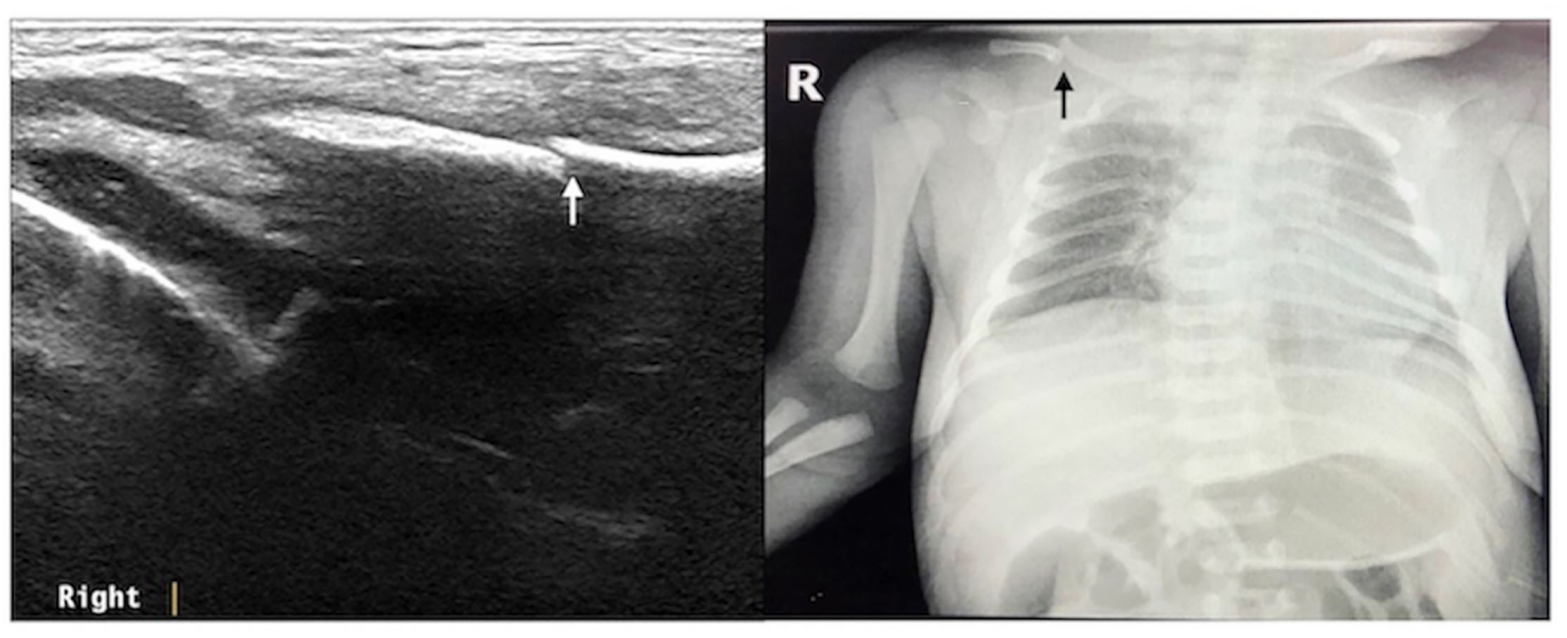
Right clavicle fracture. The infant was G_2_P_1_ with a gestational age of 39^+1^ weeks, vaginal delivery, and birth weight of 4,140 g. The patient was admitted to the hospital 3 h after birth due to intrauterine pneumonia. Physical examination showed that the bilateral clavicles were asymmetrical and that the left side was smooth, while the right side had obvious bone rubbing. Ultrasound revealed interrupted continuity of the cortical bone of the right clavicle and broken end formation and dislocation. The fracture of the clavicle was confirmed by chest X-ray examination.

**Figure 3:**
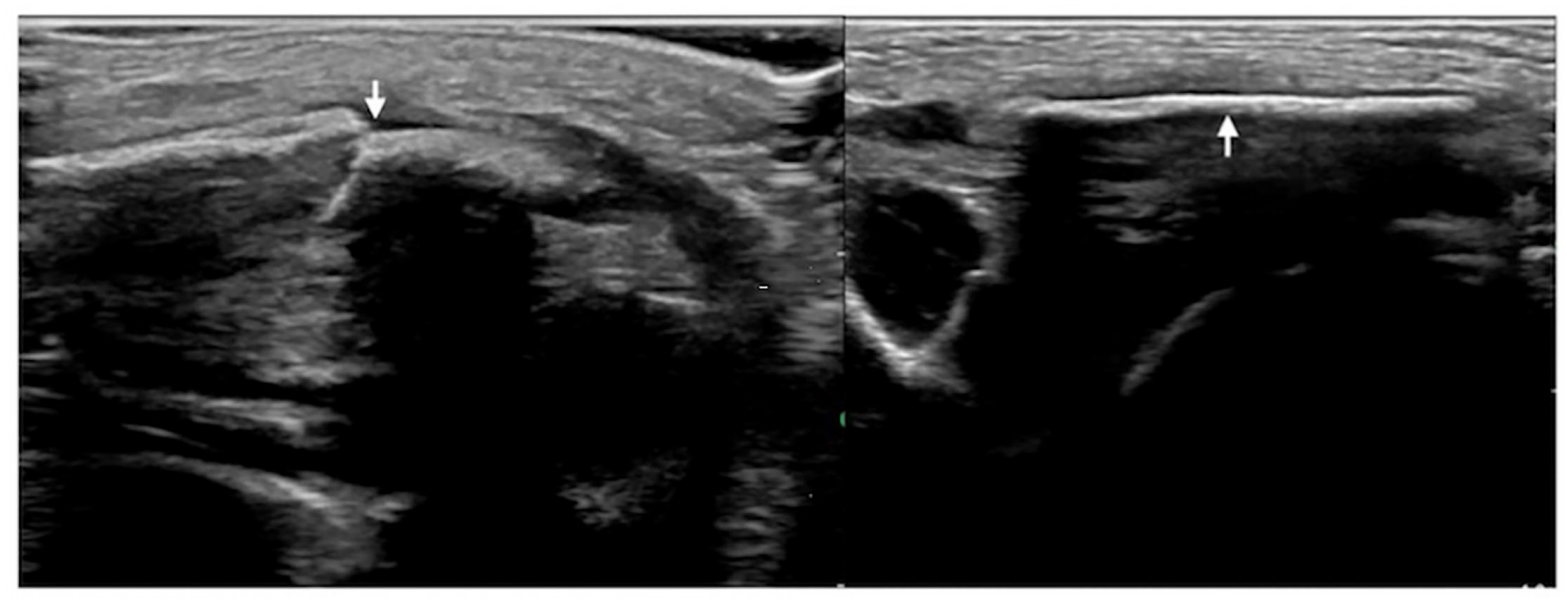
Left clavicle fracture. The infant was G_4_P_2_ with a gestational age of 41 weeks, forceps delivery, and a birth weight of 3,670 g. The patient was admitted to the hospital 41 h after birth due to jaundice for 6 h. Physical examination revealed asymmetrical clavicles as well as local swelling and bone rubbing on the left side. Ultrasound showed interrupted cortical continuity, broken end formation, and dislocation in the left midclavicular segment (left); the right clavicular continuity was intact, and the cortical bone was smooth.

**Figure 4:**
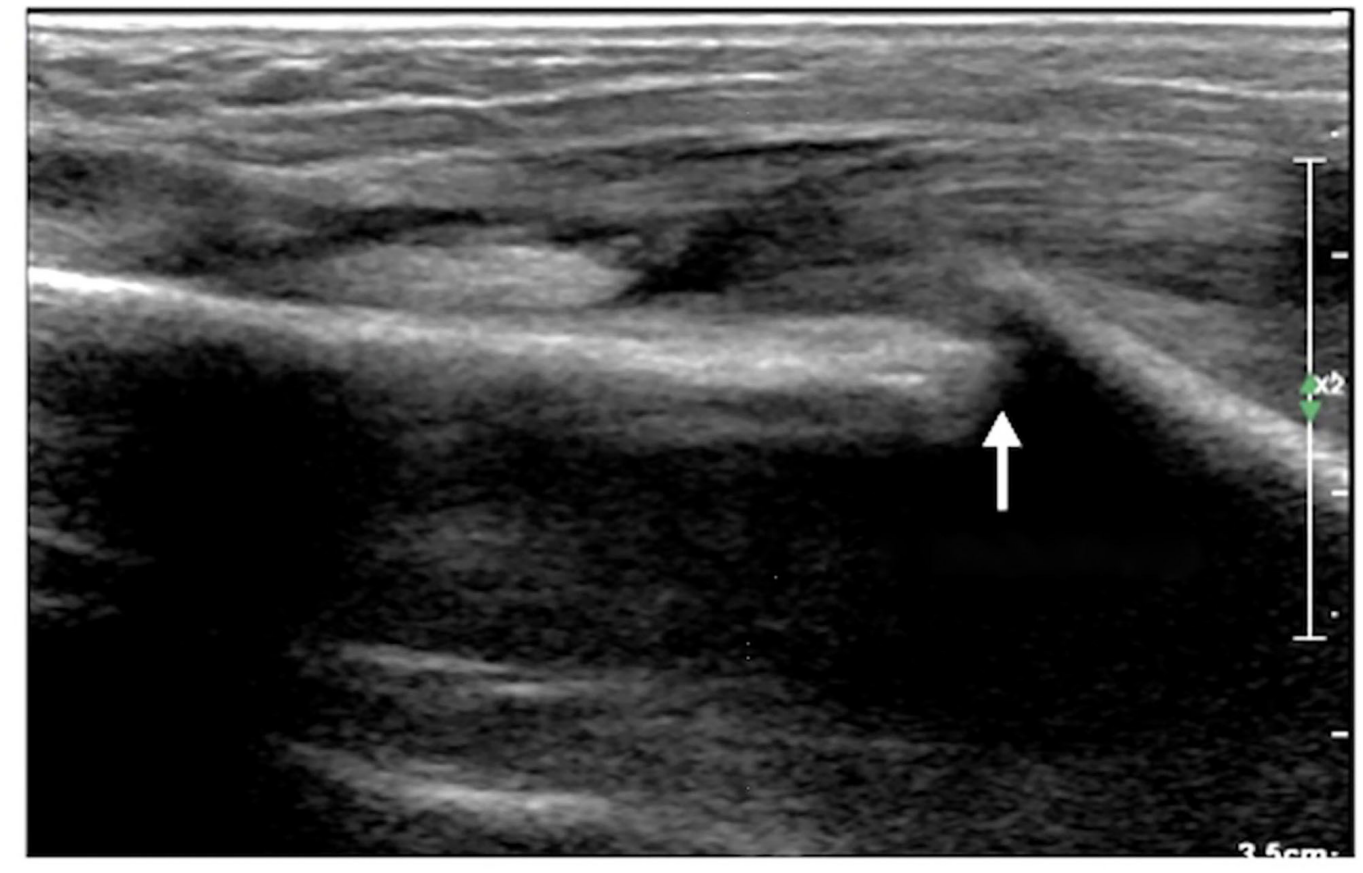
Humeral fracture. The infant was G_2_P_1_ with a gestational age of 39^+2^ weeks, forceps delivery, and birth weight of 4,000 g. The patient was admitted to the hospital 20 h after birth due to a 6-h fever. The diagnosis was intrauterine pneumonia. Physical examination revealed swelling, tenderness, limited mobility, and loss of the primitive reflex of the right upper limb; thus, the fracture was suspected. Ultrasound showed interrupted cortical continuity, visible broken ends, displacement, separation, and angulation of the right humerus.

**Figure 5:**
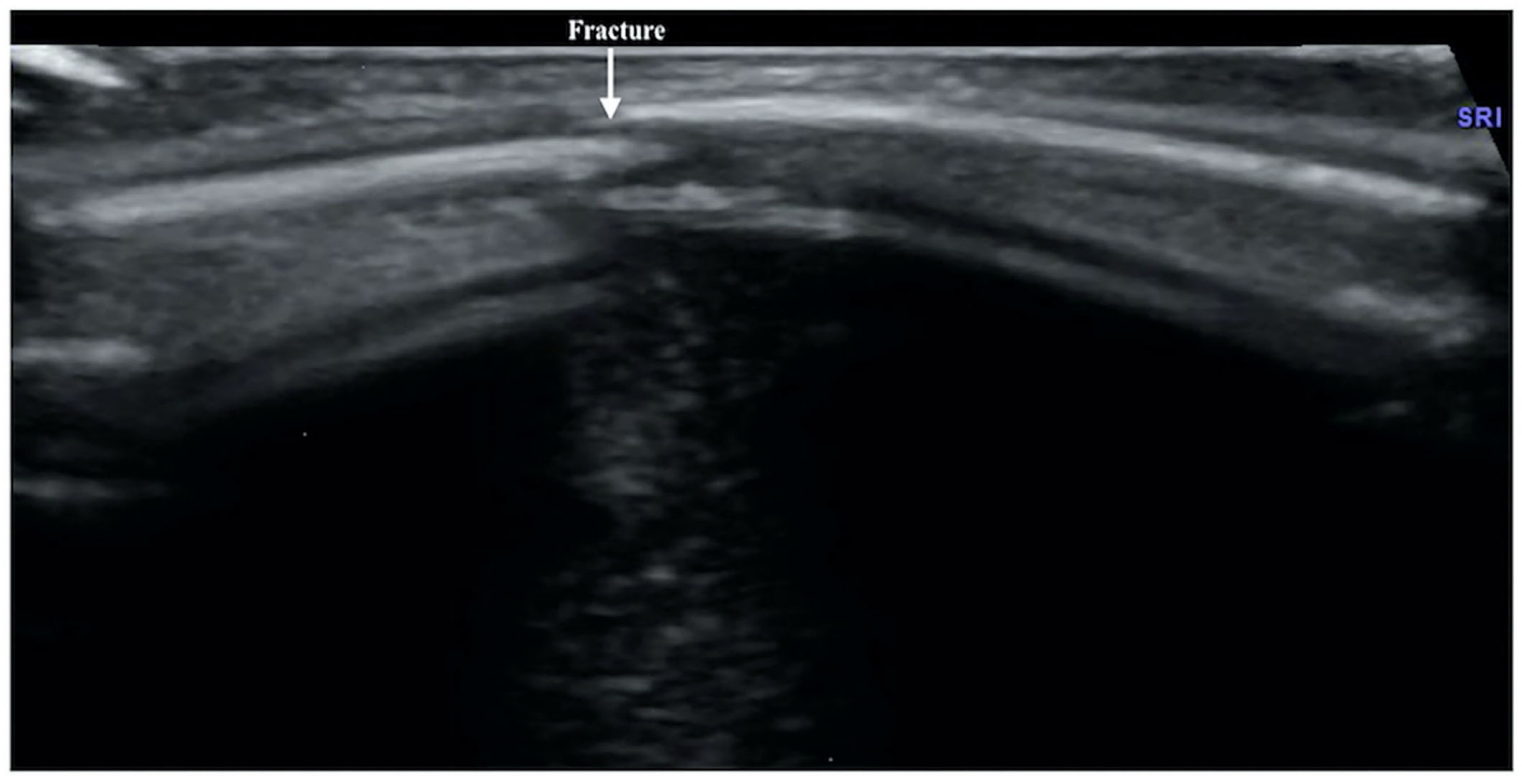
Rib fracture. The infant was G_3_P_1_, with a gestational age of 30^+1^ weeks and a birth weight of 1,370 g. The infant was born by Cesarean section due to placental abruption. After birth, the infant suffered from various diseases, such as respiratory distress syndrome, pneumonia, atelectasis, and calcium and phosphorus metabolism disorder, which was diagnosed as metabolic bone disease. Forty days after birth, an ultrasound examination found that the infant had a fracture in the fifth rib on the left side.

**Figure 6:**
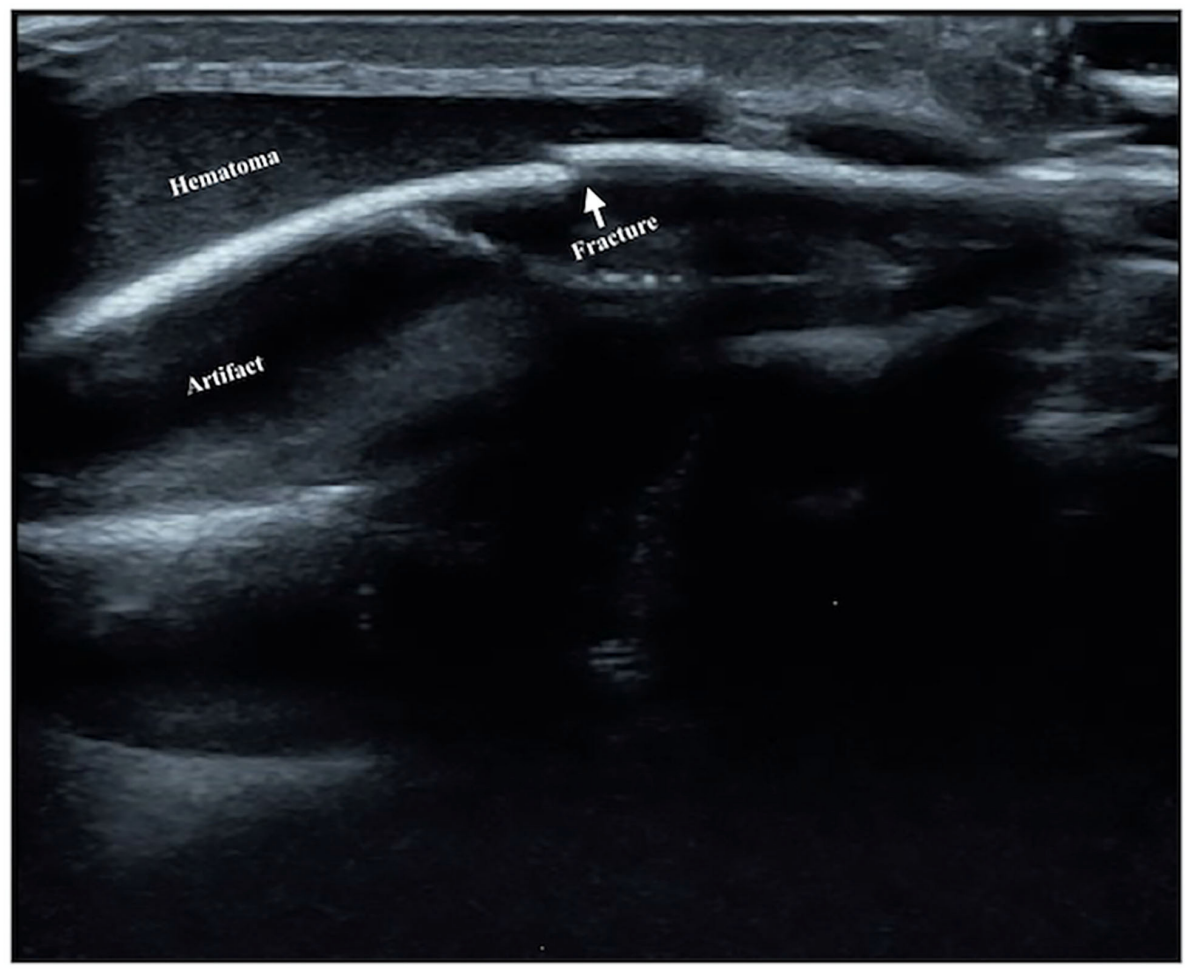
Skull fracture. The infant is G_1_P_1_ with a gestational age of 39^+2^ weeks, vaginal delivery, and birth weight of 3,940 g. He suffered from severe asphyxia at birth and was diagnosed with HIE and cranial hematoma on the top of the left head on admission. Brain ultrasound revealed that the continuity of the skull bone under the hematoma was interrupted, and the formation of broken ends with slight dislocation and separation was seen, which suggested the presence of a skull fracture.

**Figure 7:**
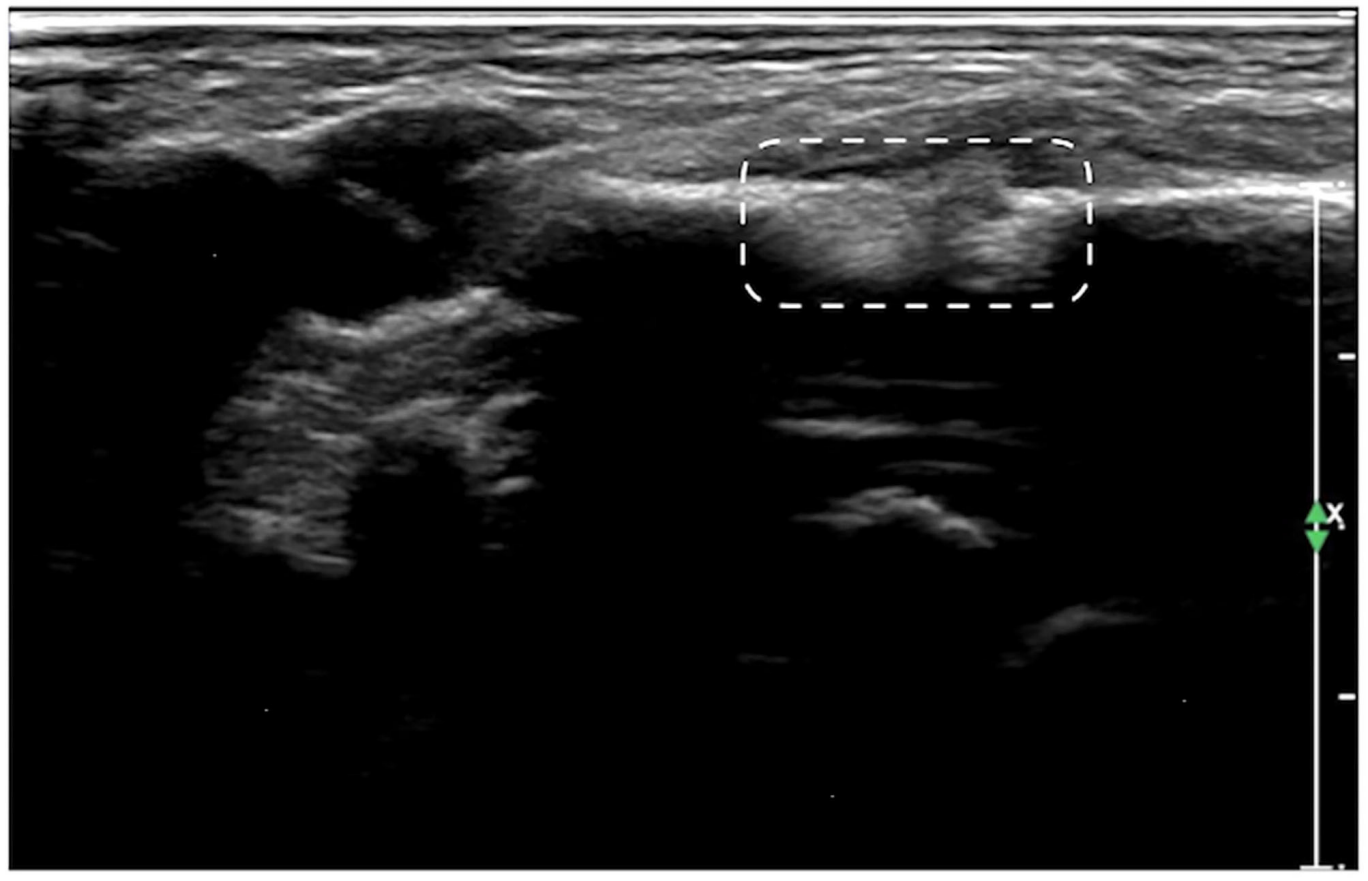
Callus formation. The infant was G_1_P_1_, with a gestational age of 39^+5^ weeks, vaginal delivery, and a birth weight of 3,700 g. The infant was admitted to the hospital on the 7th day after birth due to jaundice. Physical examination revealed local swelling and callus formation on the medial segment of the right clavicle. Ultrasound examination showed interrupted continuity and local bone tissue proliferation forming a callus at this fracture site (within the dashed line).

The ultrasound manifestations of neonatal fractures in this group included obviously interrupted cortical continuity and broken end separation at the fracture site. In severe cases, angulation or displacement could be observed, which was similar to the X-ray findings (Figures 2–6). External periosteal callus formed in the subperiosteal regions adjacent to the fracture and was observed during the recovery period (Figure 7).

### The Sensitivity and Specificity of Ultrasound for Diagnosing Fractures

**Figure 8:**
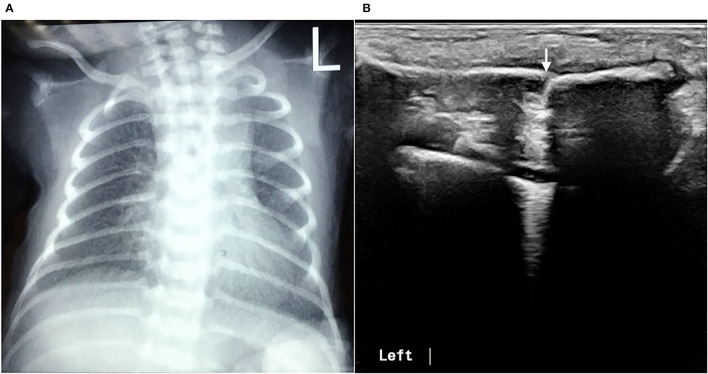
Missed clavicle fracture on X-ray. The infant underwent a chest X-ray due to postnatal dyspnea, but there were no signs of fractures in either clavicle **(A)**. Physical examination found a bone rubbing sensation on the left clavicle on admission, and the subsequent ultrasound examination confirmed the presence of a fracture of the left clavicle **(B)**.

In this study, 30 infants with clavicle fractures underwent both X-ray and ultrasound examinations. The results showed that all 30 fractures were detected by ultrasound, but one fracture was not detected by X-ray (Figure 8). Therefore, the sensitivity of ultrasound diagnosis of fractures (100%) is higher than that of X-ray (97.7%). The specificity of ultrasound for diagnosing clavicle fractures was 100%.

## Discussion

Fractures are common birth injuries in newborn infants and may include clavicle fractures, skull fractures, humerus fractures, and femur fractures. Of these, clavicle fractures are the most common; they are mainly observed in vaginally delivered infants with large birth weight and are often accompanied by shoulder dystocia. Metabolic bone disease is a rare cause of neonatal fractures and is mainly found in preterm infants (7, 8). In the present study, two premature infants with rib fractures were diagnosed with metabolic bone disease. Even rarer causes of neonatal fractures are hereditary connective tissue diseases, and particularly for cases of multiple fractures, attention should be devoted to the possibility of osteogenesis imperfecta (9).

Generally, the diagnosis of clavicular fractures mainly depends on chest X-ray examination, which can detect fracture lines. X-ray is considered the “gold standard” for fracture diagnosis. The results of this study showed that using ultrasound to diagnose fractures is more accurate and reliable than a chest X-ray. Ultrasound detected 100% of neonatal fractures, and both its sensitivity and specificity for the diagnosis of fractures were 100%. In contrast, X-ray missed one case of clavicle fracture, so its positive rate for the diagnosis of fracture was only 96.7%. With ultrasound, the scanning direction and angle can be adjusted at any time as needed and according to the position of the infant, while X-ray does not allow for adjustments. X-ray can lead to missed diagnosis of fractures (especially clavicle fractures) due to the influence of factors, such as the body position of the infant, the position of the bone at the time of imaging, the angle of the imaging or X-ray, and the alignment of the broken ends of the fracture (when the broken ends of fractures are well aligned, X-ray examinations often fail to detect them). Therefore, ultrasound is superior to X-ray for the diagnosis of neonatal fractures. The superiority of ultrasound is especially obvious for the diagnosis of neonatal rib fractures, and it can identify fractures that X-ray cannot detect (10, 11). Among the 52 patients, there were 46 cases of clavicle fracture, 3 cases of skull fracture, 2 cases of rib fracture, and 1 case of humerus fracture, and all fractures were clearly diagnosed by ultrasound. This indicates that ultrasound can not only accurately diagnose the most common clavicle fractures in clinical practice, but can also accurately diagnose relatively rare rib fractures, limb fractures, and skull fractures. In addition, X-ray is inconvenient and time-consuming, increasing radiation exposure not only to the patient himself but also to other infants within the same wards and medical staff. Therefore, the use of ultrasound for the diagnosis of neonatal fractures is of great value.

Normal cortical bone presented as hyperechogenicity on ultrasound, and the site where the hyperechogenicity was interrupted indicated the broken end of the fracture. Therefore, the main ultrasound manifestations of fractures include obviously interrupted cortical continuity, broken ends, displacement, or dislocation, and there may be an acoustic shadow behind the fractured ends. Other manifestations include local cortical upturns or bifurcations and a hypoechogenicity pattern in the surrounding soft tissues (suggesting concurrent peripheral hematoma). If a callus is formed, a hypoechoic or irregular, slightly hyperechoic pattern can be observed, and the echo intensity of the callus close to the broken ends may be close to normal and may show local fusiform enlargement (12).

Ultrasound has been used for the diagnosis of fracture, mainly in long bones and adults or children, and it is rarely used for newborn infants, especially for clavicle fracture, skull fracture, and rib fracture (13–15). To our knowledge, this is the first study to demonstrate that ultrasound can be used to diagnose common types of neonatal fractures with high accuracy and reliability in a large sample size. The diagnosis of fractures requires the use of a high-frequency linear array probe. To diagnose fractures of the clavicle, humerus, or femur, the probe is placed at a specific angle or even perpendicular to the bone being examined during ultrasound examination and is then rotated clockwise or counterclockwise. It is necessary to keep the infant in the prone position and keep the probe parallel to the ribs during scanning for the diagnosis of rib fractures to improve the positive rate of diagnosis.

There are some limitations to this study. The diagnosis of fractures with ultrasound requires certain operating skills, which require a certain period of training to master. While osteomyelitis can be missed by ultrasound due to its similar clinical presentation to that of fractures, such as local swelling, pain, and limited movement, osteomyelitis is uncommon among neonates (16–18). In addition, osteomyelitis will not have obviously interrupted cortical continuity or broken end separation at the fracture site, unless the bone affected by osteomyelitis is already broken.

In conclusion, the diagnosis of fractures by ultrasound is accurate, reliable, simple, intuitive, non-invasive, and radiation-free, and it does not require frequent changes in the infant's position, thereby reducing the possibility of aggravating the fracture or causing secondary fractures. Medical staff can master this procedure after appropriate study and training. This research will help the development and application of ultrasound diagnosis of fractures in newborns.

## Data Availability Statement

The original contributions presented in the study are included in the article/supplementary material, further inquiries can be directed to the corresponding author.

## Ethics Statement

The studies involving human participants were reviewed and approved by the Ethics Committee of Beijing Chaoyang District Maternal and Child Healthcare Hospital. Written informed consent to participate in this study was provided by the participants' legal guardian/next of kin.

## Author Contributions

JL had full access to all the data in the study and takes responsibility for the integrity of the data, the accuracy of the data analysis, manuscript preparation, and approval of the final manuscript. LZ and R-XQ contributed to the data analysis, manuscript preparation, and approval of the final manuscript. All authors contributed to the article and approved the submitted version.

## Funding

This work was supported by the Social Development Projects, Beijing Chaoyang District Bureau of Science, Technology, and Information (CYSF1922).

## Conflict of Interest

The authors declare that the research was conducted in the absence of any commercial or financial relationships that could be construed as a potential conflict of interest.

## Publisher's Note

All claims expressed in this article are solely those of the authors and do not necessarily represent those of their affiliated organizations, or those of the publisher, the editors and the reviewers. Any product that may be evaluated in this article, or claim that may be made by its manufacturer, is not guaranteed or endorsed by the publisher.
